# Comparison of Web-Based and Paper-Based Administration of ADHD Questionnaires for Adults

**DOI:** 10.2196/jmir.2225

**Published:** 2013-03-21

**Authors:** Oliver Hirsch, Franziska Hauschild, Martin H Schmidt, Erika Baum, Hanna Christiansen

**Affiliations:** ^1^Faculty of MedicineDepartment of General Practice/Family MedicinePhilipps University MarburgMarburgGermany; ^2^Faculty of PsychologyDepartment of Clinical PsychologyPhilipps University MarburgMarburgGermany

**Keywords:** computers, Attention-Deficit/Hyperactivity Disorder, questionnaires, Internet, psychometrics

## Abstract

**Background:**

Satisfactory psychometric properties in offline questionnaires do not guarantee the same outcome in Web-based versions. Any construct that is measured online should be compared to a paper-based assessment so that the appropriateness of online questionnaire data can be tested. Little research has been done in this area regarding Attention-Deficit/Hyperactivity Disorder (ADHD) in adults.

**Objective:**

The objective was to simultaneously collect paper-based and Web-based ADHD questionnaire data in adults not diagnosed with ADHD in order to compare the two data sources regarding their equivalence in raw scores, in measures of reliability, and in factorial structures.

**Methods:**

Data from the German versions of the Connors Adult ADHD Rating Scales (CAARS-S), the Wender Utah Rating Scale (WURS-k), and the ADHD Self Rating Scale (ADHS-SB) were collected via online and paper questionnaires in a cross-sectional study with convenience sampling. We performed confirmatory factor analyses to examine the postulated factor structures in both groups separately and multiple group confirmatory factor analyses to test whether the postulated factor structures of the questionnaires were equivalent across groups. With Cronbach alpha, we investigated the internal consistency of the postulated factors in the different questionnaires. Mann-Whitney U tests with the effect size “Probability of Superiority (PS)” were used to compare absolute values in the questionnaires between the two groups.

**Results:**

In the paper-based sample, there were 311 subjects (73.3% female); in the online sample, we reached 255 subjects (69% female). The paper-based sample had a mean age of 39.2 years (SD 18.6); the Web-based sample had a mean age of 30.4 years (SD 10.5) and had a higher educational background. The original four factor structure of the CAARS-S could be replicated in both samples, but factor loadings were different. The Web-based sample had significantly higher total scores on three scales. The five-factor structure of the German short form of the WURS-k could be replicated only in the Web-based sample. The Web-based sample had substantially higher total scores, and nearly 40% of the Web-based sample scored above the clinically relevant cut-off value. The three-factor structure of the ADHS-SB could be replicated in both samples, but factor loadings were different. Women in the Web-based sample had substantially higher total scores, and 30% of the Web-based sample scored above the clinically relevant cut-off value. Internal consistencies in all questionnaires were acceptable to high in both groups.

**Conclusions:**

Data from the Web-based administration of ADHD questionnaires for adults should not be used for the extraction of population norms. Separate norms should be established for ADHD online questionnaires. General psychometric properties of ADHD questionnaires (factor structure, internal consistency) were largely unaffected by sampling bias. Extended validity studies of existing ADHD questionnaires should be performed by including subjects with a diagnosis of ADHD and by randomizing them to Web- or paper-based administration.

## Introduction

Satisfactory psychometric properties in offline questionnaires do not guarantee the same outcome in Web-based versions. Any construct that is measured online should be compared to a paper-based assessment so that the appropriateness of online questionnaire data can be tested [1]. After analyzing common preconceptions about Internet questionnaires, Gosling et al [2] conclude that the quality of online data is comparable to traditional paper-and-pencil methods. The authors argue that Internet samples are not representative of the general population, but that traditional methods also do not achieve this. Web-based questionnaires are even considered to be more feasible in order to collect data in large population-based epidemiological studies [3].

Several studies did not find substantial differences between Web-based and paper-based modes of administration [4-7]. They were able to show similar psychometric properties or identical factor structures [8-10]. Those latter studies did not use clinical questionnaires to assess psychopathology, but no significant differences were found when depression questionnaires were administered in paper and online versions among the same individuals [11]. Comparable results regarding psychometric properties and absolute differences in Internet versus paper-and-pencil administration of several panic and agoraphobia questionnaires were found by Carlbring et al [12]. Both studies included pre-selected or self-recruited patient groups applying for treatment. A Web Screening Questionnaire for mental disorders yielded a high number of false positives though [13], while others reported satisfactory diagnostic accuracy in the Web-based detection of depressive disorders [14].

Buchanan [15] is skeptical of using Web-based questionnaires for normative comparisons, especially in clinical psychology. Several studies showed that score distributions between paper and online administration differed, with higher scores in Internet samples [16]. He therefore argued not to compare online questionnaire data to established norms.

Attention-Deficit/Hyperactivity Disorder (ADHD), with its core symptoms of inattention, hyperactivity, and impulsivity, is listed under disorders usually first diagnosed in childhood or adolescence in DSM-IV and ICD-10. It was shown that ADHD often persists into adulthood with prevalence rates between 4 to 5% [17-19]. We found only two studies in which measurement of ADHD via Web-based versions was examined. Steenhuis et al [20] applied a within-subject design to administer the ADHD section of the Diagnostic Interview Schedule for Children (DISC-IV) to parents. Intraclass correlation coefficients ranged between .87 and .94. A qualitative study examined acceptability of the Web-based version of the ADHD rating scale T-SKAMP in 19 teachers [21]. A large majority of teachers preferred the Web-based version over a paper version. They perceived it to be easier, shorter, simpler, and more informative, time saving, and flexible. Communication between teachers and physicians might be improved with this tool. No further ADHD diagnostic instruments, such as the SNAP and SWAN Scale for children [22] or common adult ADHD assessment instruments (see [23] for a review) were implemented as Web-based versions.

The Conners Adult ADHD Rating Scales (CAARS) [24] had satisfactory psychometric properties in their German translation [25-27] and were found to have the same factor structure as the American original, enabling them to be used for cross-cultural research. The aim of our study was to simultaneously collect paper-based and Web-based CAARS questionnaire data together with two other established ADHD questionnaires available in German and to compare the two data sources by different statistical measures regarding their equivalence in raw scores, measures of reliability, and factorial structures. To do so, we intended to collect normative data online and via paper questionnaires from subjects without a diagnosis of ADHD in order to examine whether online normative data can be merged with data from paper questionnaires.

## Methods

### Recruitment

We conducted a cross-sectional study on German adults with no serious chronic disease, who were over 18 years of age and without a lifetime diagnosis of ADHD. Participants in the paper-based sample were recruited by convenience sampling (university students, people from apprentice institutions, local neighborhoods, waiting areas such as airports, hairdressers, primary care physicians, and colleagues). Subjects were provided with a short study description and asked to complete the CAARS self-report (CAARS-S) as well as the German version of the Wender Utah Rating Scale (WURS-k), the German ADHD Self Rating Scale (ADHS-SB), and questions on age, gender, and education level. We disseminated approximately 500 printed questionnaires.

The Web-based questionnaire was also a cross-sectional convenience sample. We advertised for the online study on the websites of the Departments of General Practice/Family Medicine and Clinical Psychology at Philipps University Marburg and on a special Facebook page created exclusively for our study. Additionally, flyers with the online address of the Web-based questionnaire were distributed in the same recruitment areas as the paper-based questionnaires. For informed consent in the online study, the homepage prompted subjects to open a file with the study information and to check a box agreeing to participate in the study. Without checking this box, further pages of the questionnaire were not accessible. Since the survey was voluntary, all subjects had the ability to discontinue completing the questionnaire at any time. Subjects could see their progress in completing the questionnaire via a small progress bar on the upper right side of the screen. On average, subjects needed 15:34 minutes to complete the survey; the majority of participants completed the survey during the afternoon (hour 15, or 3 p.m.). At the end of the questionnaire, subjects had the opportunity to receive feedback on their responses. This indicated whether their scores were within the normal range or higher. In cases of the latter, no diagnosis was offered, but it was suggested to seek professional assessment. Data protection was insured in that only the principal investigator (HC) had access to the unipark page [28] that generated and stored the data. Additionally, no personal information was requested of the subjects.

For development and testing, the paper versions of the questionnaires were entered into unipark. The research team and then students were asked to test this online version. The link was activated after testing for functionality and usability.

The survey was online from July 12, 2010, to August 30, 2011. On average, the page was accessed 26.16 times per week (view rate), though only 6.69 (25 %) subjects per week completed the survey (completion rate). Cookies were used to assign a unique user identifier to each client computer and were set on the first page. A session was valid for a total of 120 minutes.

**Figure 1:**
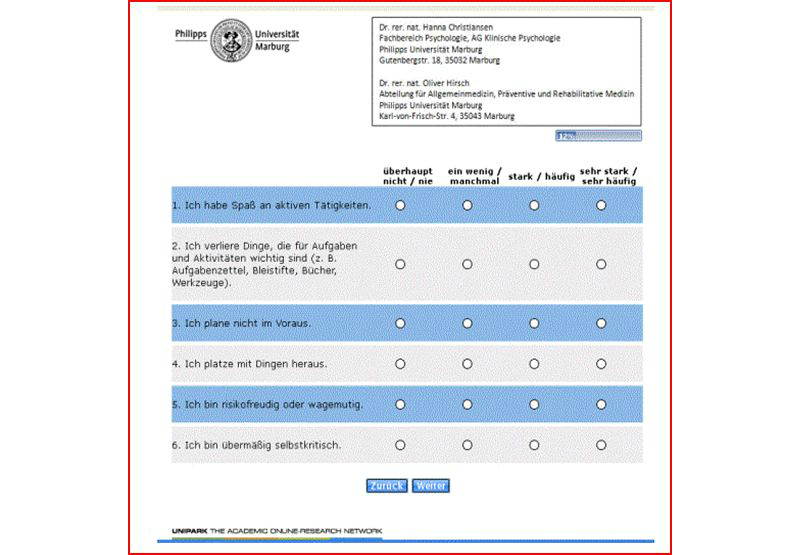
Example of survey items per page.

The items were presented in the same order as the paper-and-pencil questionnaires, but only an average of six items were displayed per page (see Figure 1). When subjects did not complete all items on a page, they were asked to fill in the missing items in order to activate the “next” button. Therefore, no missing data could result in the online sample. Subjects were always able to review and change their answers with a “back” button. If subjects decided not to complete an answer, they could stop the survey. The majority of participants (57.44 %) discontinued filling out the survey on the first page.

For analyses, only questionnaires where subjects indicated they had not received a lifetime diagnosis of ADHD were analyzed. Apart from replacing missing items in paper versions with the expectation-maximization or the multiple imputation algorithms, no statistical corrections were performed.

Our study conforms to the Declaration of Helsinki and was approved by the local ethics committee of the Faculty of Medicine at the Philipps University in Marburg, Germany.

### Measurements

#### Connors Adult ADHD Rating Scales

The German version of the CAARS-S assesses ADHD symptoms in adults aged 18 years or older. Symptoms are rated on a Likert-type scale (0 = *not at all/never* to 3 = *very much/very frequently*). The long version consists of 66 items, but only 42 items were included in the original factor analysis by Conners et al [24] due to statistical restrictions made by the authors. Four factors emerged from their analyses: inattention/memory problems, hyperactivity/restlessness, impulsivity/emotional ability, and problems with self-concept. Confirmatory factor analyses of the German version in healthy adults and ADHD patients supported this factor analytic solution [25,27]. The four subscales were significantly influenced by age, gender, and the number of years in education. Symptom severity decreased with increasing age, males scored higher than females on hyperactivity and sensation-seeking behavior, and females scored higher than males on problems with self-concept. Overall symptom ratings were higher for individuals who had received less education. Test-retest reliability ranged between .85 and .92; sensitivity and specificity were high for all four subscales. The CAARS-S represents a reliable and cross-culturally valid measure of current ADHD symptoms in adults [26].

#### Wender Utah Rating Scale

The German version of the Wender Utah Rating Scale (WURS-k) [29,30] retrospectively assesses ADHD-relevant childhood behaviors and symptoms in adults. It consists of 25 items that distinguished patients with ADHD from a nonpatient comparison group. Subjects are instructed to rate 25 items that complete sentence stems such as ‘‘As a child I was or had...’’. Ratings are to be completed on a 5-point Likert scale (0 =*not at all or very slightly* to 4=*very much*). Test-retest reliability and Cronbach alpha were around .90. Factor analyses generated a 5-factor solution with the factors inattention/hyperactivity, impulsivity, anxiety/depression, oppositional behavior, and social adaptation by using 21 items. A total score >29 points hints at the possibility of ADHD during childhood.

#### ADHD Self Rating Scale

The German ADHD Self Rating Scale (ADHS-SB) consists of the 18 DSM-IV items that are broken down into the factors “inattention” (9 items), “hyperactivity”, and “impulsivity” (9 items together) [31]. The items are scored on a 4-point Likert scale (0=*not at all* to 3=*very pronounced/almost always the case*). Test-retest reliability coefficients were between .78 and .89. Correlations with subscales of the NEO Five Factor Inventory were in the expected directions. A total score >17 points hints at the possibility of adult ADHD.

### Statistical Analysis

We performed confirmatory factor analyses to examine the postulated factor structures in both groups separately and multiple group confirmatory factor analyses using AMOS 19 to test whether the postulated factor structures of the questionnaires were equivalent across the groups. The factors were allowed to correlate because this is theoretically plausible in all three questionnaires. We used unweighted least squares as this estimation method makes no distributional assumptions [32].

Using multiple group analysis, we examined several levels of invariance between the groups. Configural invariance as the lowest level of invariance exists when the structure of the factor loading matrices is identical in all groups. Metric invariance occurs when factor loadings are identical in all groups. Scale invariance means that the measurement intercepts are the same across groups. Invariance of measurement errors exists if the error variables of measurement models, factor covariances, and factor variances are identical across groups.

We calculated several model fit indices to evaluate the results of our analyses. The root mean square residual (RMR) measures the mean absolute value of the covariance residuals [33]. Values less than .05 indicate a good model fit [32], but other authors state that a value of less than .10 signals an acceptable model fit [34-35]. The standardized root mean square residual (SRMR) eliminates scaling effects of the RMR. Values ≤ .10 indicate a good model fit [35]. The Global Fit Index (GFI) can measure the proportion of variance and covariance that a given model is able to explain. A GFI equal or higher than .90 can be considered as reflecting a good model fit [36]. The adjusted global fit index (AGFI) takes the number of parameters used in computing the GFI into account. An AGFI equal or higher than .90 can be considered as showing a good model fit [35]. These fit indices were calculated for each of the aforementioned invariance levels. Differences of fit indices between these invariance levels should not be larger than .01 [35], otherwise the criteria for a higher invariance level are not reached.

With Cronbach alpha, we investigated the internal consistency of the postulated factors in the different questionnaires. Values >.70 are considered to be acceptable [37].

Huber’s M estimators were calculated when standard deviation values were close to their respective means, signaling high variance [38].

Mann-Whitney U tests were used to compare absolute values in the questionnaires between the two groups. The effect size “Probability of Superiority (PS)”, PS=U/(n1*n2), indicates the probability that a randomly selected subject of group n1 has a higher score than a randomly selected subject of group n2. A PS of .50 means that both groups are equal regarding a specific variable, and that there is no effect. Consequently, the larger the effect, the more PS deviates from .50 [39].

The alpha level for statistical significance was set at .05 (two-sided). Missing responses in the paper versions were replaced using the expectation-maximization or the multiple imputation algorithms [40-42].

## Results

### Samples

**Figure 2:**
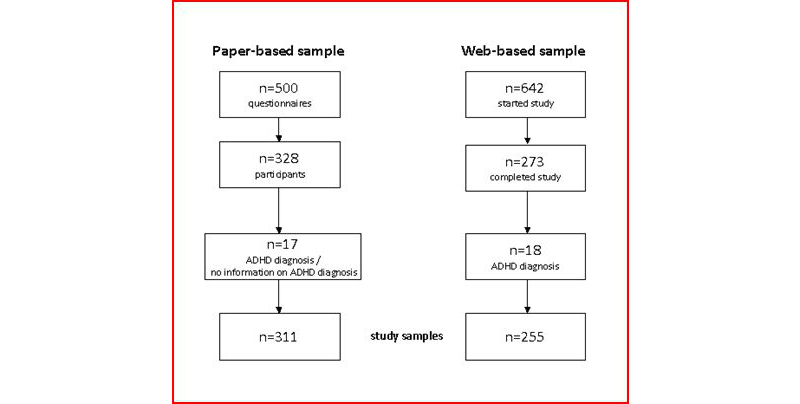
Flow of subjects in the paper-based and Web-based samples.

In the paper-based sample, we received responses from 328 participants of which 6 indicated they were diagnosed with ADHD, and 11 did not answer this question. Therefore, a total sample of 311 subjects resulted, meaning that 65.6% of our 500 printed questionnaires were returned. This cannot be regarded as a return rate as we did not record those subjects who were personally asked and refused to participate. In the Web-based sample, we received responses from 273 participants of which 18 indicated that they were diagnosed with ADHD so that a total sample of 255 subjects resulted. The flow of subjects in our study samples is depicted in Figure 2. Table 1 shows the demographic characteristics of the two samples.

**Table 1:** Demographic characteristics of the paper-based and Web-based samples.

		Paper-based (n=311)	Web-based (n=255)
**Gender**			
	Female	228 (73.3%)	176 (69.0%)
	Male	83 (26.7%)	79 (31.0%)
**Age, mean years (SD)**		39.2 (18.6)	30.4 (10.5)
**Education**			
	University	61 (19.7%)	73 (28.6%)
	Apprenticeship	86 (27.7%)	35 (13.7%)
	High school	66 (21.3%)	130 (51.0%)
	Middle school	61 (19.7%)	15 (5.9%)
	Basic school	36 (11.6%)	2 (0.8%)

The samples did not differ with respect to gender (χ^2^ test: *P*=.26, Cramer V=.05). The Web-based sample was, on average, younger than the paper-based sample. This difference was statistically significant with a rather moderate effect size (Mann-Whitney U test: *P*<.001, PS = .41). In the Web-based sample, there were more participants with a university degree and more subjects attending high school, while in the paper-based sample, there were more participants attending middle or basic school or with a completed apprenticeship. This difference was statistically significant with a high effect size (χ^2^ test: *P*<.001, Cramer V=.42).

#### Connors Adult ADHD Rating Scales

There was a maximum of 9% missing values on single variables in the paper sample; these were missing completely at random (Little’s MCAR test, *P*=.27). They were replaced with the expectation maximization (EM) algorithm [40].

The four-factor model (df = 813) was supported in both groups. In the paper-based sample, the standardized RMR was .08, the RMR was .04, the GFI was .93, and the AGFI was .92. In the Web-based sample, the standardized RMR was .07, the RMR was .05, the GFI was .98, and the AGFI was .97. These fit indices signal a good model fit. Table 2 lists the correlations of CAARS items (loadings) with their postulated factors.

Except for Item 3 (“I don’t plan ahead”) of the factor “inattention/memory”, Items 1 (“I like to be doing active things”) and 5 (“I am a risk-taker or a daredevil”) of the hyperactivity factor, and Item 43 (“I step on people’s toes without meaning to”) on the impulsivity factor, all other items have loadings > .40 in both samples.

The intercorrelations between the factors are consistently higher in the Web-based sample. The largest differences between the two groups were found in correlations involving “self-concept” (see Table 3).

Multiple group analysis revealed that the factor structures were the same in both samples, signaling configural invariance (SRMR=.04, RMR=.04, GFI=.99, AGFI=.99). However, factor loadings were different (SRMR=.06, RMR=.10, GFI=.97, AGFI=.97) because all model fit indices increased > .01 when testing metric invariance. Consequently, other invariance assumptions were also not supported.

Cronbach alpha of the subscales ranged from .81 to .85 in the paper-based sample and from .89 to .91 in the Web-based sample.

Absolute subscale differences between the two groups were all significant with the Web-based sample scoring substantially higher with pronounced effect sizes (Table 4). For example, the probability that a randomly chosen subject from the paper-based sample has a higher inattention/memory score than a randomly chosen subject from the Web-based sample is .35.

As there is no normative data for Germany to date, we applied strict cut-off values based on American normative data T-value of 65, 94th percentile). The cut-off for “inattention/memory” was > 22 points; “hyperactivity” > 26 points; “impulsivity” > 22 points; and “self-concept” > 13 points. Regarding the total score of “inattention/memory”, 27 subjects (10.6%) in the Web-based sample scored above this value while 4 (1.3%) did so in the paper-based sample. This difference was significant with a moderate effect size (χ^2^ test: *P*<.001, Cramer V=.20). Regarding the total score of “hyperactivity”, 5 subjects (2.0%) in the Web-based sample scored above this value while 2 (0.6%) did so in the paper-based sample. This difference was not significant (χ^2^ test: *P*=.16, Cramer V=.06). Regarding the total score of “impulsivity”, 20 subjects (7.8%) in the Web-based sample scored above this value while 6 (1.9%) did so in the paper-based sample. This difference was significant with a small effect size (χ^2^ test: *P*=.001, Cramer V=.14). Regarding the total score of “self-concept”, 30 (11.8%) in the Web-based sample scored above this value while 13 (4.2%) did so in the paper-based sample. This difference was significant with a small effect size (χ^2^ test: *P*=.001, Cramer V=.14).

#### Wender Utah Rating Scale (Short Form)

There was a maximum of 2% missing values on single variables in the paper sample that were missing completely at random (Little’s MCAR test, *P*=.57). These were replaced with the EM algorithm [40]. Due to technical difficulties, WURS-k data of 11 participants in the Web-based sample were not available, resulting in n=244.

The model in the paper-based sample was not admissible because the covariance matrix between the postulated five factors was not positive definite. This leads to the conclusion that the model is wrong [34], and it was thus rejected.

In the Web-based sample, the model (df = 179) was supported: SRMR = .07, RMR = .09, GFI = .98, AGFI = .97. Table 5 depicts the loadings on the postulated factors.

As shown in Table 5, except for Item 23 (problems with police) on the factor “social adaptation”, all other items have high loadings > .4 on their postulated factors. The factors “inattention” and “impulsivity” correlated highest in the Web-based sample (r=.79), followed by “inattention” and “anxiety/depression” (r=.73), “impulsivity” and “oppositional behavior” (r=.72), and “inattention” and “oppositional behavior” (r=.72) (Table 6).

Due to the rejected model in the paper-based group, no multiple group analysis could be calculated.

Cronbach alpha of the subscales ranged from .68 to .82 in the paper-based sample and from .79 to .89 in the Web-based sample. No coefficients were calculated for the subscale “social adaptation” as it consists of only two items.

Absolute values of the total score were significantly higher (Mann-Whitney U test: *P*<.001; PS = .09) in the Web-based sample (mean 28.6, SD 14.0; Huber’s M estimator 26.2) than in the paper-based sample (mean 11.0, SD 6.8; Huber’s M estimator 9.6).

After applying the recommended cut-off value for the total score (> 29 points) [29,30], 38.1% in the Web-based sample scored above this value while merely 2.5% did so in the paper-based sample. This difference was significant with a high effect size (χ^2^ test: *P*<.001, Cramer V=.46).

#### ADHD Self Rating Scale

There was a maximum of 1.3% missing values on single variables in the paper sample, except for Item 4 that asks for difficulties in the field of work. Student participants in the paper version did not complete this item, so 28.5% of missing at random data resulted. These were replaced with the multiple imputation algorithm by five imputations. The following calculations were done separately for the five imputations, and the respective results were averaged. Enders [40] recommends a larger number of imputations, but results showed only marginal differences between imputed datasets.

The four-factor model (df = 132) was supported in both groups. In the paper-based sample, the standardized RMR was .06, the RMR was .06, the GFI was .97, and the AGFI was .96. In the Web-based sample, the standardized RMR was .06, the RMR was .04, the GFI was .98, and the AGFI was .98. These fit indices signal a good model fit. Table 7 lists the loadings of ADHS-SB items on their postulated factors.

As shown in Table 7, except for Item 6 (avoidance of tasks with mental load) on the factor “inattention”, and Item 14 (feel like driven by a motor) on the factor “hyperactivity”, both in the paper-based sample, all other items have high loadings >.4 on their postulated factors.

The correlation between the factors inattention and hyperactivity is significantly higher in the paper-based sample, while the intercorrelations between the other factors are higher in the Web-based sample (Table 8).

Multiple group analysis revealed that the factor structures were the same in both samples, signaling configural invariance (SRMR = .06, RMR = .03, GFI = .98, AGFI = .98). However, factor loadings were different (SRMR = .15, RMR = .08, GFI = .83, AGFI = .79) because all model fit indices increased > .01 when testing metric invariance. Consequently, other invariance assumptions were also not supported.

Cronbach alpha of the subscales ranged from .60 to .83 in the paper-based sample and from .79 to .91 in the Web-based sample.

Absolute differences between the two groups were significant with the Web-based sample (mean 12.8, SD 9.1; Huber’s M estimator 11.1) scoring substantially higher than the paper-based sample (mean 2.2, SD 3.0; Huber’s M estimator 1.4) with a high effect size (Mann-Whitney U test: *P*<.001, PS = .08).

After applying the recommended cut-off value for the total score (> 17 points) [31], 30% in the Web-based sample scored above this value, while only 0.6% did so in the paper-based sample. This difference was significant with a large effect size (χ^2^ test: *P*<.001, Cramer V=.42).

**Table 2:** Correlations of CAARS items (loadings) with their postulated factors (latent constructs) in the paper-based and Web-based samples.

		Paper-based	Web-based
**Inattention/Memory**			
	ITEM 03	.14	.26
	ITEM 07	.53	.74
	ITEM 11	.59	.73
	ITEM 16	.47	.67
	ITEM 18	.55	.74
	ITEM 32	.57	.61
	ITEM 36	.69	.75
	ITEM 40	.52	.73
	ITEM 44	.55	.78
	ITEM 49	.55	.73
	ITEM 51	.55	.63
	ITEM 66	.57	.74
**Hyperactivity**			
	ITEM 01	.31	.06
	ITEM 05	.43	.32
	ITEM 10	.46	.50
	ITEM 13	.70	.74
	ITEM 20	.65	.69
	ITEM 25	.57	.46
	ITEM 27	.57	.82
	ITEM 31	.65	.66
	ITEM 38	.54	.76
	ITEM 46	.67	.80
	ITEM 54	.61	.71
	ITEM 57	.73	.81
**Impulsivity**			
	ITEM 04	.56	.62
	ITEM 08	.49	.72
	ITEM 12	.56	.69
	ITEM 19	.66	.58
	ITEM 23	.58	.61
	ITEM 30	.64	.76
	ITEM 35	.47	.54
	ITEM 39	.60	.67
	ITEM 43	.37	.59
	ITEM 47	.59	.78
	ITEM 52	.53	.62
	ITEM 61	.61	.69
**Self-concept**			
	ITEM 06	.59	.58
	ITEM 15	.60	.75
	ITEM 26	.58	.69
	ITEM 37	.81	.86
	ITEM 56	.75	.79
	ITEM 63	.81	.84

**Table 3:** Intercorrelations between the CAARS factors (latent constructs) in the paper-based and Web-based samples.

Factors			Paper-based	Web-based
Hyperactivity	<-->	Impulsivity	.73	.81
Inattention/Memory	<-->	Hyperactivity	.54	.74
Inattention/Memory	<-->	Impulsivity	.65	.79
Inattention/Memory	<-->	Self-concept	.47	.74
Hyperactivity	<-->	Self-concept	.24	.57
Impulsivity	<-->	Self-concept	.45	.71

**Table 4:** Means, standard deviations, and Huber’s M estimators of the CAARS subscales in the paper-based and Web-based samples with their respective *P* and effect size values.

	Paper-based	Web-based	Mann-Whitney U Test (*P*) & effect size (PS^b^)
Inattention/Memory	8.6 (SD 4.8) Huber’s M^a^ 8.2	12.3 (SD 7.1) Huber’s M 11.2	<.001; PS=.35
Hyperactivity	9.0 (SD 5.3) Huber’s M 8.0	11.2 (SD 6.1) Huber’s M 10.3	<.001; PS=.38
Impulsivity	9.4 (SD 5.2) Huber’s M 8.8	12.3 (SD 6.6) Huber’s M 11.5	<.001; PS=.37
Self-concept	5.6 (SD 3.6) Huber’s M 5.1	7.5 (SD 4.3) Huber’s M 7.1	<.001; PS=.37

^a^Huber’s M estimator.

^b^PS = probability of superiority.

**Table 5:** Correlations of WURS-k items (loadings) with their postulated factors (latent constructs) in the Web-based sample.

		Web-based
**Inattention**		
	ITEM 01	.82
	ITEM 02	.76
	ITEM 03	.78
	ITEM 06	.77
	ITEM 10	.75
	ITEM 15	.59
	ITEM 17	.67
	ITEM 24	.51
**Impulsivity**		
	ITEM 05	.75
	ITEM 11	.83
	ITEM 13	.83
	ITEM 16	.89
**Anxiety/Depression**		
	ITEM 07	.74
	ITEM 09	.62
	ITEM 18	.74
	ITEM 19	.80
**Oppositional behavior**		
	ITEM 08	.88
	ITEM 21	.54
	ITEM 22	.79
**Social adaptation**		
	ITEM 20	.64
	ITEM 23	.33

**Table 6:** Intercorrelations between the WURS-k factors (latent constructs) in the Web-based sample.

Factors			Web-based
Inattention	<-->	Impulsivity	.79
Impulsivity	<-->	Anxiety/Depression	.69
Inattention	<-->	Anxiety/Depression	.73
Inattention	<-->	Social adaptation	.50
Impulsivity	<-->	Oppositional behavior	.72
Impulsivity	<-->	Social adaptation	.49
Anxiety/Depression	<-->	Oppositional behavior	.35
Anxiety/Depression	<-->	Social adaptation	.57
Oppositional behavior	<-->	Social adaptation	.58
Inattention	<-->	Oppositional behavior	.72

**Table 7:** Correlations of ADHS-SB items (loadings) with their postulated factors (latent constructs) in the paper-based and Web-based samples.

		Paper-based	Web-based
**Inattention**			
	ITEM 01	.47	.72
	ITEM 02	.48	.73
	ITEM 03	.56	.68
	ITEM 04	.40	.65
	ITEM 05	.45	.63
	ITEM 06	.22	.62
	ITEM 07	.48	.54
	ITEM 08	.60	.72
	ITEM 09	.45	.64
**Hyperactivity**			
	ITEM 10	.71	.74
	ITEM 11	.66	.69
	ITEM 12	.58	.82
	ITEM 13	.54	.65
	ITEM 14	.31	.59
**Impulsivity**			
	ITEM 15	.68	.72
	ITEM 16	.49	.72
	ITEM 17	.48	.73
	ITEM 18	.58	.58

**Table 8:** Intercorrelations between the ADHS-SB factors (latent constructs) in the paper-based and Web-based samples.

Factors			Paper-based	Web-based
Inattention	<-->	Hyperactivity	.92	.63
Hyperactivity	<-->	Impulsivity	.64	.80
Inattention	<-->	Impulsivity	.66	.72

## Discussion

We compared Web-based and paper-based administrations of three ADHD questionnaires for adults. Subjects in the online sample were older and had a higher educational background. The original four-factor structure of the Conners Adult ADHD Rating Scales could be replicated in both samples, but factor loadings were different. Internal consistencies were high in both groups, but the Web-based sample had significantly higher total scores in three subscales with 7.8 to 11.8% above clinically relevant cut-off values, compared to 1.3 to 4.2% in the paper-based sample. The five-factor structure of the German short form of the Wender Utah Rating Scale could be replicated only in the Web-based sample. Internal consistencies were acceptable to high in both groups. The Web-based sample had substantially higher total scores and nearly 40% of the Web-based sample scored above the clinically relevant cut-off value. The three-factor structure of the ADHD Self Rating Scale could be replicated in both samples, but factor loadings were different. Internal consistencies were acceptable to high in both groups. The Web-based sample had substantially higher total scores, and 30% of the Web-based sample scored above the clinically relevant cut-off value. Therefore, psychometric properties were similar in both samples, but the Web-based sample had substantially higher scores on all three questionnaires.

The relatively high dropout rate in our Web-based sample is also reported in the literature. Additional informed consent procedures were shown to increase early dropout in Web-based studies [43]. Kongsved et al [44] administered paper and online questionnaires to women referred for mammography; their questionnaires were comparable in length to our study. In their study, the Internet version had a higher completeness of data but a lower response rate. A lower response rate was also found in surgeons responding to an online questionnaire [45]. We cannot compare dropout rates in our samples because we did not record those who were personally asked and refused to participate in the paper-based sample.

Demographic differences (younger age, higher education) might have influenced the results [46]. ADHD is a disorder with a higher prevalence for men. Our predominantly female samples in both versions are therefore not suitable for deriving normative data. Women tend to participate more in psychological studies. The gender distributions in our study are quite comparable to common sample characteristics regarding online study participation [2,47]. Subjects in the Internet sample might have experienced psychological distress regarding ADHD symptoms so that they saw their participation in the context of assessing themselves for the disorder. One also has to consider that the probability that subjects in a certain geographic region have a high prevalence of ADHD symptoms in paper-based questionnaires is much lower than the probability of reaching such individuals via the Internet without any geographic barriers. On the other hand, an increased self-disclosure might also be an important factor. Subjects might have considered the completion of online questionnaires to be more anonymous than giving away hand-written information, since participants in an online study reported lower social anxiety and lower social desirability than those in the paper-based group [48].

Our results contradict the conclusion of Gosling et al [2] that subjects in Internet samples are not unusually maladjusted. It clearly depends on the context of the study. Even when the intention is to collect normative data for clinical questionnaires, one has to consider that the scores of online samples can be inflated [49].

On the other hand, our results corroborate the assumption of Rhodes et al [47] that previously hidden subgroups can be reached by Internet research. This might also be true for other medical disorders [50]. Whether our subgroup of women with higher scores on ADHD questionnaires has clinical significance must be determined by future studies with more controlled recruitment strategies.

Several limitations have to be mentioned. We did not randomize subjects to online and paper versions, so differences between the two groups might have arisen by sampling biases and should be replicated under randomized conditions. Different recruitment strategies for the paper and online samples might have influenced the results. Although relatively high discontinuation rates are common in online research, they might have caused bias in the results. In future online studies, leaving out questions should also be possible to create conditions similar to paper administration.

### Conclusions

Data from the Web-based administration of ADHD questionnaires for adults should not be used for the extraction of population norms. Separate norms should be established for ADHD online questionnaires. General psychometric properties of ADHD questionnaires (factor structure, internal consistency) were largely unaffected by sampling bias. Extended validity studies of existing ADHD questionnaires should be performed by including subjects with a diagnosis of ADHD and by randomizing them to Web- or paper-based administration.

## Abbreviations

ADHD: Attention-Deficit/Hyperactivity Disorder
ADHS-SB: ADHD Self Rating Scale
AGFI: Adjusted Global Fit Index
CAARS: Conners Adult ADHD Rating Scales
CAARS-S: CAARS self-report
DSM-IV: Diagnostic and Statistical Manual of Mental Disorders-Fourth Edition
GFI: Global Fit Index
ICD-10: International Classification of Diseases—Tenth Edition
MCAR: Missing completely at random
PS: Probability of Superiority
RMR: Root Mean Square Residual
SRMR: Standardized Root Mean Square Residual
WURS-k: Wender Utah Rating Scale-Short Form
